# Supplementary material for: Sustained-input switches for transcription factors and microRNAs are central building blocks of eukaryotic gene circuits
Source: Genome Biol. 2013 Aug 23;14(8):R85. doi: 10.1186/gb-2013-14-8-r85 (PMC4054853; doi:10.1186/gb-2013-14-8-r85)
Supplement: Additional file 5 — HTML Browsable Motif Output. Zipped folder containing all WaRSwap and FANMOD motif output, viewable in a web browser. [file gb-2013-14-8-r85-S5.ZIP › HTML_browsable_motif_output/WaRSwap_all_tair9/sigs_WSWRSLDraw-2000_all.pvals.heatmaps.html/motif_id_46_000101110_tftype_all_upstream_-1000_0.html]

```
BG_MODEL = Rnew2
MOTIF_ID = 46_000101110
TF_TYPE = all
UPSTREAM = -1000_0


PVals
FN_0.2	FN_0.4	FN_0.6	FN_0.8
dg_60.genes	0.072	0.002	0.006	0.053
dg_70.genes	0.065	0.003	0.006	0.051
dg_80.genes	0.064	0.001	0.006	0.05

ZScores
FN_0.2	FN_0.4	FN_0.6	FN_0.8
dg_60.genes	1.471	2.644	2.642	1.698
dg_70.genes	1.448	2.549	2.562	1.734
dg_80.genes	1.49	2.563	2.562	1.754

StDevs
FN_0.2	FN_0.4	FN_0.6	FN_0.8
dg_60.genes	1570.631	403.456	58.173	3.097
dg_70.genes	1609.574	409.72	58.267	3.033
dg_80.genes	1580.929	415.926	58.855	3.04
```
